# Supplementary material for: Commodity-specific triads in the Dutch inter-industry production network
Source: Sci Rep. 2024 Feb 13;14:3625. doi: 10.1038/s41598-024-53655-3 (PMC10864404; doi:10.1038/s41598-024-53655-3)
Supplement: Supplementary file 1 — Supplementary Information. [file 41598_2024_53655_MOESM1_ESM.pdf]

Supplementary Information for "Commodity-specific triads in the Dutch inter-industry production network"

Marzio Di Vece[1,2,3*], Frank P. Pijpers[4,5], and Diego Garlaschelli[1,2,6]

[1]IMT School for Advanced Studies Lucca, P.zza San Francesco 19, 55100 Lucca (Italy)
[2]Lorentz Institute for Theoretical Physics, Leiden University, Niels Bohrweg 2, 2333CA Leiden (The Netherlands)
[3]Scuola Normale Superiore, P.zza dei Cavalieri 7, Pisa (Italy)
[4]Statistics Netherlands, Henri Faasdreef 312, 2492 JP Den Haag (the Netherlands)
[5]Korteweg - de Vries Institute for Mathematics, University of Amsterdam, Amsterdam (the Netherlands)
[6]INdAM-GNAMPA Istituto Nazionale di Alta Matematica (Italy)
[*]marzio.divece@imtlucca.it

ABSTRACT

This supplementary information goes into detail of the construction of our inter-industry multi-commodity production network from the dataset collected by Statistics Netherlands. The second part discusses the Maximum Log-likelihood estimation of the statistical null models and the sampling of links and weights.

Dataset

The description of the CBS production network for 2018 is rephrased from the article[1]. For completeness, we rephrase here which are the key micro-data ingredients, used to build it. Firm-level data is obtained from the General Business Register (ABR) for 2018, containing data for over 1 700 000 firms. After cleaning for micro-firms with annual net turnover below 10 000€, around 900 000 firms remain, accounting for 99.5% of the Dutch economy output in 2018. The breakdown in commodities is extracted from the Structural Business Statistics (SBS) survey for commercial industries, from the Prodcom survey for manufacturing industries, and estimated by National Accounts for non-commercial industries. In most cases, the commodity breakdown data is available for the industry as a whole and not for individual organizations within industries. A breakdown of intermediate supply and use per firm follows using intermediate purchases as the distributional key, and then an ulterior breakdown in commodity groups is performed by integrating data from the SBS and Prodcom surveys. The resulting dataset is then compared to the industry-level supply/use tables at the SBI4 classification, and appropriate rescaling of supply and use per firm is performed by Iterative Proportion Fitting. Once supply and use per firm per commodity are obtained, their in-degree distribution is estimated using stylized facts from[2] Japanese firms. Suppliers and users are then matched according to a deterministic procedure that takes into account (1) their trade capacity, encoded by their net turnover, (2) their mutual distance, (3) the presence of a link between respective industries, (4) the observed relationship in the Dun & Bradstreet dataset, which contains the customers of the largest 500 suppliers in the Dutch Economy. Finally, the resulting inter-firm network at the 650 commodity level is compared to the inter-industry (known) network at the 250 commodity level and consequent adjustments are done to weights and links. Due to the known biases in the deterministic imputation procedure affecting network density and degree sequences[3] the dataset does not represent a reliable *ground-truth* as it is.

To reduce the bias, we take advantage of the tested coherence between the inter-firm network for 192 commodities and the inter-industry network as the key point of our pre-processing. Specifically, we aggregate the 650 commodity groups into 192, coherently with industry-level known tables, and then we aggregate firms according to their SBI5 Standard Industry Classification extracted from the ABR for 2018. Passing from the SBI4 to the SBI5 classification leads to a greater granularity in industry resolution, increasing the number of industries from 132 to 870. Finally, we clean for intra-industry trade and obtain a multi-layer inter-industry production network containing linkages and weights for 862 industries (nodes) and 187 commodity groups (layers). For the topic of interest, triadic motifs, the self-loops implied by intra-industry trade are not important and can be removed from the dataset without adversely affecting the subsequent analysis. Therefore, although intra-industry trade is certainly relevant for both intensive and extensive margins (weights and links respectively) they are ignored.

Estimation of Binary Null Models

The Directed Binary Configuration Model

The Directed Binary Configuration Model (DBCM)[4] is the maximum-entropy model where the out-degree and in-degree sequences are constrained. The corresponding Graph Hamiltonian is

$$H(A) = \sum_i \left(\alpha_i^{out} k_i^{out} + \alpha_i^{in} k_i^{in}\right) = \sum_{i,j\neq i} \left(\alpha_i^{out} + \alpha_j^{in}\right) a_{ij}. \tag{1}$$

The partition function reads

$$Z(A) = \sum_A e^{-H(A)} = \sum_A e^{-\sum_{i,j\neq i}\left(\alpha_i^{out}+\alpha_j^{in}\right)a_{ij}} = \prod_{i,j\neq i} \sum_{a_{ij}=0,1} \left(x_i^{out} x_j^{in}\right)^{a_{ij}} = \prod_{i,j\neq i} \left(1 + x_i^{out} x_j^{in}\right) \tag{2}$$

where $x_i^y = e^{-\alpha_i^y}$ with $\cdot = \{out, in\}$.

After computing the partition function the binary graph distribution $P(A)$ is

$$P(A) = \frac{e^{-H(A)}}{Z(A)} = \prod_{i,j\neq i} \frac{\left(x_i^{out} x_j^{in}\right)^{a_{ij}}}{1 + x_i^{out} x_j^{in}} \tag{3}$$

It is possible to define a Log-Likelihood from $P(A)$ as

$$\mathscr{L} = \ln P(A) = -H(A) - \ln(Z(A)) = -\sum_i \left(\alpha_i^{out} k_i^{out} + \alpha_i^{in} k_i^{in}\right) - \sum_{i,j\neq i} \ln(1 + x_i^{out} x_j^{in}). \tag{4}$$

Parameters are then estimated using MLE on the log-likelihood function which consists in solving the following set of equations for the node-specific parameters $\alpha_i^{out}, \alpha_i^{in}$.

$$\begin{cases} \dfrac{\partial \mathscr{L}}{\partial \alpha_i^{out}} & = -k_i^{out} + \sum_{j\neq i} \left(\dfrac{x_i^{out} x_j^{in}}{1 + x_i^{out} x_j^{in}}\right); \\ \dfrac{\partial \mathscr{L}}{\partial \alpha_i^{in}} & = -k_i^{in} + \sum_{j\neq i} \left(\dfrac{x_i^{in} x_j^{out}}{1 + x_i^{in} x_j^{out}}\right). \end{cases} \tag{5}$$

The Reciprocal Binary Configuration Model

The Reciprocal Binary Configuration Model (RBCM)[6] is the maximum-entropy model constraining the reciprocated and non-reciprocated degree sequences. The corresponding Graph Hamiltonian reads

$$H(A) = \sum_i (\alpha_i^{\rightarrow} k_i^{\rightarrow} + \alpha_i^{\leftarrow} k_i^{\leftarrow} + \alpha_i^{\leftrightarrow} k_i^{\leftrightarrow}) = \sum_{i,j<i} \left(\alpha_i^{\rightarrow} + \alpha_j^{\leftarrow}\right) a_{ij}^{\rightarrow} + \left(\alpha_i^{\leftarrow} + \alpha_j^{\rightarrow}\right) a_{ij}^{\leftarrow} + \left(\alpha_i^{\leftrightarrow} + \alpha_j^{\leftrightarrow}\right) a_{ij}^{\leftrightarrow} \tag{6}$$

The model-induced partition function is

$$\begin{aligned} Z(A) &= \sum_A e^{-H(A)} = \sum_A \prod_{i,j<i} \left(x_i^{\rightarrow} x_j^{\leftarrow}\right)^{a_{ij}^{\rightarrow}} \left(x_i^{\leftarrow} x_j^{\rightarrow}\right)^{a_{ij}^{\leftarrow}} \left(x_i^{\leftrightarrow} x_j^{\leftrightarrow}\right)^{a_{ij}^{\leftrightarrow}} = \prod_{i,j<i} \sum_{\{a_{ij}\}} \left(x_i^{\rightarrow} x_j^{\leftarrow}\right)^{a_{ij}^{\rightarrow}} \left(x_i^{\leftarrow} x_j^{\rightarrow}\right)^{a_{ij}^{\leftarrow}} \left(x_i^{\leftrightarrow} x_j^{\leftrightarrow}\right)^{a_{ij}^{\leftrightarrow}} = \\ &= \prod_{i,j<i} \left(1 + x_i^{\rightarrow} x_j^{\leftarrow} + x_i^{\leftarrow} x_j^{\rightarrow} + x_i^{\leftrightarrow} x_j^{\leftrightarrow}\right) \end{aligned} \tag{7}$$

where $x_i^y = e^{-\alpha_i^y}$ with $y = \{\rightarrow, \leftarrow, \leftrightarrow\}$, and the last equality arises because by definition the events described by different arrows - e.g. $a_{ij}^{\rightarrow}$, $a_{ij}^{\leftarrow}$, $a_{ij}^{\leftrightarrow}$ and $a_{ij}^{\not\leftrightarrow}$ - are mutually exclusive, i.e. only one of those terms is equal to 1 while all the others are equal to zero for each dyad (i,j).

After computing the partition function the binary Graph Probability $P(A)$ is obtained as follows

$$P(A) = \frac{e^{-H(A)}}{Z(A)} = \prod_{i,j<i} \frac{\left(x_i^{\rightarrow} x_j^{\leftarrow}\right)^{a_{ij}^{\rightarrow}} \left(x_i^{\leftarrow} x_j^{\rightarrow}\right)^{a_{ij}^{\leftarrow}} \left(x_i^{\leftrightarrow} x_j^{\leftrightarrow}\right)^{a_{ij}^{\leftrightarrow}}}{1 + x_i^{\rightarrow} x_j^{\leftarrow} + x_i^{\leftarrow} x_j^{\rightarrow} + x_i^{\leftrightarrow} x_j^{\leftrightarrow}} \tag{8}$$

From the partition function, we define the log-likelihood function $\mathscr{L}$

$$\begin{aligned}\mathscr{L} &= \ln P(A) = -H(A) - \ln(Z(A)) = \\ &= -\sum_i (\alpha_i^{\rightarrow} k_i^{\rightarrow} + \alpha_i^{\leftarrow} k_i^{\leftarrow} \alpha_i^{\leftrightarrow} k_i^{\leftrightarrow}) - \sum_{i,j<i} \ln(1 + x_i^{\rightarrow} x_j^{\leftarrow} + x_i^{\leftarrow} x_j^{\rightarrow} + x_i^{\leftrightarrow} x_j^{\leftrightarrow})\end{aligned} \tag{9}$$

and we solve for the node-specific set of parameters $\{\alpha_i^{\rightarrow}, \alpha_i^{\leftarrow}, \alpha_i^{\leftrightarrow}\}$ by using the MLE framework

$$\begin{cases} \dfrac{\partial \mathscr{L}}{\partial \alpha_i^{\rightarrow}} &= -k_i^{\rightarrow} + \sum_{j\neq i} \left(\dfrac{x_i^{\rightarrow} x_j^{\leftarrow}}{1 + x_i^{\rightarrow} x_j^{\leftarrow} + x_i^{\leftarrow} x_j^{\rightarrow} + x_i^{\leftrightarrow} x_j^{\leftrightarrow}} \right); \\ \dfrac{\partial \mathscr{L}}{\partial \alpha_i^{\leftarrow}} &= -k_i^{\leftarrow} + \sum_{j\neq i} \left(\dfrac{x_i^{\leftarrow} x_j^{\rightarrow}}{1 + x_i^{\rightarrow} x_j^{\leftarrow} + x_i^{\leftarrow} x_j^{\rightarrow} + x_i^{\leftrightarrow} x_j^{\leftrightarrow}} \right); \\ \dfrac{\partial \mathscr{L}}{\partial \alpha_i^{\leftrightarrow}} &= -k_i^{\leftrightarrow} + \sum_{j\neq i} \left(\dfrac{x_i^{\leftrightarrow} x_j^{\leftrightarrow}}{1 + x_i^{\rightarrow} x_j^{\leftarrow} + x_i^{\leftarrow} x_j^{\rightarrow} + x_i^{\leftrightarrow} x_j^{\leftrightarrow}} \right). \end{cases} \tag{10}$$

Binary Sampling

Networks are sampled according to the DBCM recipe by (1) computing the induced connection probability $p_{ij;DBCM}$ and (2) establishing a link between industry i and j if and only if a uniformly distributed random number $u_{ij} \in U(0,1)$ is below $p_{ij;DBCM}$. The analogous recipe for RBCM requires (1) computing the set of connection probabilities for non-reciprocated connection between i and j, namely $p_{ij}^{\rightarrow}$, $p_{ij}^{\leftarrow}$ and $p_{ij}^{\not\leftrightarrow}$, and reciprocated connection $p_{ij}^{\leftrightarrow}$, generate a uniform random variable $u_{ij} \in (0,1)$ and (2) establishing the appropriate links in the dyad in the following way:

- a non-reciprocated link from i to j if $u_{ij} \leq p_{ij}^{\rightarrow}$;
- a non-reciprocated link from j to i if $u_{ij} \in (p_{ij}^{\rightarrow}, p_{ij}^{\rightarrow} + p_{ij}^{\leftarrow}]$;
- a reciprocated link from i to j (and from j to i) if $u_{ij} \in (p_{ij}^{\rightarrow} + p_{ij}^{\leftarrow}, p_{ij}^{\rightarrow} + p_{ij}^{\leftarrow} + p_{ij}^{\leftrightarrow}]$;
- no links from i to j and from j to i otherwise.

Estimation of Conditional Weighted Null Models

Conditional Reconstruction Method A

The Conditional Reconstruction Method A (CReMa)[5] is the conditional maximum-entropy model constraining the out-strength and in-strength sequences.

The Graph Hamiltonian reads

$$H(W) = \sum_i \left(\beta_i^{out} s_i^{out} + \beta_i^{in} s_i^{in}\right) = \sum_{i,j\neq i} \left(\beta_i^{out} + \beta_j^{in}\right) w_{ij}. \tag{11}$$

It induces a partition function of the following form

$$Z(W_A) = \int_{W_A} \prod_{i,j\neq i} e^{-\left(\beta_i^{out} + \beta_j^{in}\right) w_{ij}} dw_{ij} = \left(\frac{1}{\beta_i^{out} + \beta_j^{in}} \right)^{a_{ij}}. \tag{12}$$

The conditional graph probability function $Q(W|A)$ is then defined as

$$Q(W|A) = \prod_{i,j\neq i} \left[(\beta_i^{out} + \beta_j^{in}) e^{-(\beta_i^{out} + \beta_j^{in}) w_{ij}} \right]^{a_{ij}} \tag{13}$$

and the corresponding log-likelihood $\mathscr{L}$ is

$$\mathscr{L} = -\sum_i \left(\beta_i^{out} s_i^{out} + \beta_i^{in} s_i^{in}\right) + \sum_{i,j\neq i} a_{ij} \ln(\beta_i^{out} + \beta_j^{in}) \tag{14}$$

In order to take into account the random variability of the binary adjacency matrix A we average the log-likelihood over the ensemble realizations and obtain the generalized log-likelihood $\mathscr{G}$, defined as

$$\mathscr{G} = -\sum_i \left(\beta_i^{out} s_i^{out} + \beta_i^{in} s_i^{in}\right) + \sum_{i,j\neq i} p_{ij} \ln(\beta_i^{out} + \beta_j^{in}) \tag{15}$$

The node-specific parameters $\{\beta_i^{out}, \beta_i^{in}\}$ are tuned according to the first order equation for GLE, i.e.

$$\begin{cases} \dfrac{\partial \mathscr{G}}{\partial \beta_i^{out}} &= -s_i^{out} + \sum_{j \neq i} \dfrac{p_{ij}}{\beta_i^{out} + \beta_j^{in}} \\ \dfrac{\partial \mathscr{G}}{\partial \beta_i^{in}} &= -s_i^{in} + \sum_{j \neq i} \dfrac{p_{ji}}{\beta_i^{in} + \beta_j^{out}} \end{cases} \tag{16}$$

which amounts to equating the empirical and model-induced out-strength and in-strength sequences.

Conditionally Reciprocal Weighted Configuration Model

The Conditionally Reciprocal Weighted Configuration Model (or CRWCM) is a novel conditional maximum-entropy model that constrains out-strength and in-strength dividing them according to the character of the reciprocity in the underlying links. The constraints are non-reciprocated out-strengths and in-strength - $s_i^{\rightarrow}$ and $s_i^{\leftarrow}$ - and reciprocated out-strengths and in-strengths, namely $s_i^{\leftrightarrow,out}$ and $s_i^{\leftrightarrow,in}$.

The corresponding Graph Hamiltonian reads

$$H(W) = \sum_i (\beta_i^{\rightarrow} s_i^{\rightarrow} + \beta_i^{\leftarrow} s_i^{\leftarrow}) + \left(\beta_i^{\leftrightarrow,out} s_i^{\leftrightarrow,out} + \beta_i^{\leftrightarrow,in} s_i^{\leftrightarrow,in}\right) = \sum_{i,j \neq i} h(w_{ij}) \tag{17}$$

where

$$h(w_{ij}) = \left(\beta_i^{\rightarrow} + \beta_j^{\leftarrow}\right) a_{ij}^{\rightarrow} w_{ij} + \left(\beta_i^{\leftrightarrow,out} + \beta_j^{\leftrightarrow,in}\right) a_{ij}^{\leftrightarrow} w_{ij}. \tag{18}$$

The Hamiltonian induces a conditional partition function defined as

$$Z(W_A) = \int_{W_A} e^{-\sum_{i,j \neq i} h(w_{ij})} dw_{ij} = \prod_{i,j \neq i} \int_0^{\infty} e^{-h(w_{ij})} dw_{ij} = \prod_{i,j \neq i} Z_{ij|A} \tag{19}$$

where the dyadic-specific conditional partition function $Z_{ij|A}$ is

$$Z_{ij|A} = \left(\frac{1}{\beta_i^{\rightarrow} + \beta_j^{\leftarrow}}\right)^{a_{ij}^{\rightarrow}} \left(\frac{1}{\beta_i^{\leftrightarrow,out} + \beta_j^{\leftrightarrow,in}}\right)^{a_{ij}^{\leftrightarrow}} \tag{20}$$

so that $Z_{ij|A} = (\beta_i^{\rightarrow} + \beta_j^{\leftarrow})^{-1}$ if $a_{ij}^{\rightarrow} = 1$ and $Z_{ij|A} = (\beta_i^{\leftrightarrow,out} + \beta_j^{\leftrightarrow,in})^{-1}$ if $a_{ij}^{\leftrightarrow} = 1$.

The conditional probability distribution for the weighted network is

$$Q(W|A) = \prod_{i,j \neq i} \frac{e^{-h(w_{ij})}}{Z_{ij|A}}. \tag{21}$$

It induces a log-likelihood $\mathscr{L}$ of the form

$$\mathscr{L} = \sum_{i,j \neq i} \left[-h(w_{ij}) + \ln(Z_{ij|A})\right] \tag{22}$$

We take into account the random variability of A, induced by the choice of the binary model, by formulating the average log-likelihood, i.e. the generalized log-likelihood $\mathscr{G}$. It is defined as

$$\mathscr{G} = \sum_{i,j \neq i} \left[-h(w_{ij}) + \ln(Z_{ij|P(A)})\right] = \mathscr{G}^{\rightarrow} + \mathscr{G}^{\leftrightarrow} \tag{23}$$

where

$$\begin{cases} \mathscr{G}^{\rightarrow} &= \sum_{i,j \neq i} \left[-(\beta_i^{\rightarrow} + \beta_j^{\leftarrow}) w_{ij} + p_{ij}^{\rightarrow} \ln(\beta_i^{\rightarrow} + \beta_j^{\leftarrow})\right] \\ \mathscr{G}^{\leftrightarrow} &= \sum_{i,j \neq i} \left[-(\beta_i^{\leftrightarrow,out} + \beta_j^{\leftrightarrow,in}) w_{ij} + p_{ij}^{\leftrightarrow} \ln(\beta_i^{\leftrightarrow,out} + \beta_j^{\leftrightarrow,in})\right], \end{cases} \tag{24}$$

i.e. $\mathscr{G}$ can be decoupled into a *non-reciprocated* component $\mathscr{G}^{\rightarrow}$ and a *reciprocated* component $\mathscr{G}^{\leftrightarrow}$. This implies that the resulting GLE problem can be divided into two subproblems. The non-reciprocated problem equates to solving a system of $2N$ coupled equations, namely

$$\begin{cases} \frac{\partial \mathscr{G}}{\partial \beta_i^{\rightarrow}} & = -s_i^{\rightarrow} + \sum_{j\neq i} \frac{p_{ij}^{\rightarrow}}{\beta_i^{\rightarrow} + \beta_j^{\leftarrow}} \\ \frac{\partial \mathscr{G}}{\partial \beta_i^{\leftarrow}} & = -s_i^{\leftarrow} + \sum_{j\neq i} \frac{p_{ij}^{\leftarrow}}{\beta_i^{\leftarrow} + \beta_j^{\rightarrow}}. \end{cases} \tag{25}$$

The reciprocated subproblem equates to solving the following set of $2N$ coupled equations

$$\begin{cases} \frac{\partial \mathscr{G}}{\partial \beta_i^{\leftrightarrow,out}} & = -s_i^{\leftrightarrow,out} + \sum_{j\neq i} \frac{p_{ij}^{\leftrightarrow}}{\beta_i^{\leftrightarrow,out} + \beta_j^{\leftrightarrow,in}} \\ \frac{\partial \mathscr{G}}{\partial \beta_i^{\leftrightarrow,in}} & = -s_i^{\leftrightarrow,in} + \sum_{j\neq i} \frac{p_{ij}^{\leftrightarrow}}{\beta_i^{\leftrightarrow,in} + \beta_j^{\leftrightarrow,out}}. \end{cases} \tag{26}$$

Weight Sampling

In operative terms, using a two-step model such as the DBCM+CReMa reduces to (1) establishing a link between industries i and j when a uniform random number $u_{ij} \in U(0,1)$ is such that $u_{ij} \leq p_{ij;DBCM}$, (2) if i and j are connected, sampling w_{ij} by using the inverse transform sampling method technique, i.e., we generate a uniformly distributed random variable $\eta_{ij} \in U(0,1)$ such that

$$F(v_{ij}) = \int_0^{v_{ij}} q_{CReMa}(w_{ij}|a_{ij}=1)dw_{ij} = \eta_{ij}, \tag{27}$$

then we invert the relationship finding the weight v_{ij} to load on the link (i,j).

The network sampling for the RBCM+CRWCM follows the same concepts with two major differences: (1) a link is established using the RBCM recipe and (2) the dyadic conditional weight probability $q_{CReMa}(w_{ij}|a_{ij}=1)$ is substituted with $q_{CRWCM}(w_{ij}|a_{ij}=1)$ in the inverse transform sampling.

References

1. G. Buiten, E. Jonge, G. Mooijen, S. Hooijmaaijers, and P. Bogaart, OECD Conference, New Analytical Tools and Techniques for Economic Policymaking (2021).
2. H. Watanabe, H. Takayasu, and M. Takayasu, *Physica A: Statistical Mechanics and its Applications* **392**, 741 (2013).
3. A. Rachkov, F. Pijpers, and D. Garlaschelli, CBS Technical Reports 10.13140/RG.2.2.31861.29925 (2021).
4. T. Squartini, G. Fagiolo, and D. Garlaschelli, *Physical Review E* **84**, 046117 (2011).
5. F. Parisi, T. Squartini, and D. Garlaschelli, *New Journal of Physics* **22**, 053053 (2020).
6. T. Squartini and D. Garlaschelli, *New Journal of Physics* **13**, 083001 (2011).
